# High-Value, Cost-Conscious Care Attitudes in the Graduate Medical Education Learning Environment: Various Stakeholder Attitudes That Residents Misjudge

**DOI:** 10.1007/s11606-020-06261-8

**Published:** 2020-11-02

**Authors:** Serge B. R. Mordang, Andrea N. Leep Hunderfund, Frank W. J. M. Smeenk, Laurents P. S. Stassen, Karen D. Könings

**Affiliations:** 1 Department of Educational Development and Research, School of Health Professions Education, Maastricht University, Maastricht, The Netherlands; 2 Department of Neurology, Mayo Clinic, Rochester, MN USA; 3 Department of Pulmonary Medicine, Catharina Hospital, Eindhoven, The Netherlands; 4 Department of Surgery, Maastricht University Medical Center, Maastricht, The Netherlands

**Keywords:** attitudes, graduate medical education, high-value, cost-conscious care, learning environment, job characteristics

## Abstract

**Background:**

Training residents in delivering high-value, cost-conscious care (HVCCC) is crucial for a sustainable healthcare. A supportive learning environment is key. Yet, stakeholders’ attitudes toward HVCCC in residents’ learning environment are unknown.

**Objective:**

We aimed to measure stakeholders’ HVCCC attitudes in residents’ learning environment, compare these with resident perceptions of their attitudes, and identify factors associated with attitudinal differences among each stakeholder group.

**Design:**

We conducted a cross-sectional survey across the Netherlands between June 2017 and December 2018.

**Participants:**

Participants were 312 residents, 305 faculty members, 53 administrators, and 1049 patients from 66 (non)academic hospitals.

**Main Measures:**

Respondents completed the Maastricht HVCCC Attitude Questionnaire (MHAQ), containing three subscales: (1) high-value care, (2) cost incorporation, (3) perceived drawbacks. Additionally, resident respondents estimated the HVCCC attitudes of other stakeholders, and answered questions on job demands and resources. Univariate and multivariate analyses were used to analyze data.

**Key Results:**

Attitudes differed on all subscales: faculty and administrators reported more positive HVCCC attitudes than residents (*p* ≤ 0.05), while the attitudes of patients were less positive (*p* ≤ 0.05). Residents underestimated faculty’s (*p* < 0.001) and overestimated patients’ HVCCC attitudes (*p* < 0.001). Increasing age was, among residents and faculty, associated with more positive attitudes toward HVCCC (*p* ≤ 0.05). Lower perceived health quality was associated with less positive attitudes among patients (*p* < 0.001). The more autonomy residents perceived, the more positive their HVCCC attitude (*p* ≤ 0.05).

**Conclusions:**

Attitudes toward HVCCC vary among stakeholders in the residency learning environment, and residents misjudge the attitudes of both faculty and patients. Faculty and administrators might improve their support to residents by more explicitly sharing their thoughts and knowledge on HVCCC and granting residents autonomy in clinical practice.

**Electronic supplementary material:**

The online version of this article (10.1007/s11606-020-06261-8) contains supplementary material, which is available to authorized users.

## INTRODUCTION

Rising healthcare costs, overuse of care, and wasted spending demand physicians who are trained to provide high-value, cost-conscious care (HVCCC).^1–3^ HVCCC requires weighing the benefits and harms of procedures and interventions, while also considering cost over time. Physicians are expected to estimate the value of care and provide cost-effective care.^4^ Practicing HVCCC can lead to better outcomes, reduced costs, and improved patient experience.^5–7^ Residency training is an opportune time to shape future physicians’ behavior.^8–10^ A supportive learning environment is crucial,^11, 12^ as workplace experiences form residents’ future knowledge, attitudes, and behaviors.^13–16^

Learning environments are complex, dynamic phenomena that are shaped by various stakeholders.^17^ Faculty are residents’ primary role models,^18^ provide residents with advice and feedback,^19, 20^ and are the main initiators of HVCCC discussions.^21^ Administrators play an important role in policy and financial issues.^22, 23^ Patients also form an integral part of residents’ training and are increasingly involved in decision-making.^24–26^ Each stakeholder group brings a unique perspective to the issue of value and costs. For instance, patients often feel uncomfortable discussing costs of care,^27^ while administrators are under pressure to control costs.^28^ Understanding the attitudes of these important stakeholder groups toward HVCCC can thus provide valuable insights into the residency learning environment.

Attitudes feed into behavior in practice,^29, 30^ as demonstrated by studies connecting specific physician attitudes with their usage of healthcare services.^31–33^ Stakeholders in the workplace exhibit their own attitudes and show their culture, beliefs, and behaviors through the hidden curriculum.^34, 35^ Social pressure arising from the hidden curriculum can have powerful effects on residents’ attitudes and, possibly, their future behaviors.^29, 36, 37^ Hence, if a resident sees how a key stakeholder propagates HVCCC, the resident’s intend to perform HVCCC behaviors will be formed, either positively or negatively affecting the resident’s attitude toward HVCCC. Understanding how residents perceive the HVCCC attitudes of influential stakeholders within the learning environment can thus provide insights into the hidden curriculum.

Specific individual beliefs and work contexts also shape attitudes toward HVCCC, habitual behaviors, and perceptions regarding benefits and drawbacks of HVCCC.^38, 39^ Therefore, different variables, like demographics or training regions,^15, 40^ could bolster different beliefs regarding HVCCC and help clarify differences in HVCCC attitudes within stakeholder groups. Furthermore, residents’ perceptions of job characteristics may affect their willingness to devote energy and apply their skills to their work.^41^ Job demands, aspects at work that call for physical or mental exertion, can invoke stress and exhaustion to perform a behavior, while job resources, aspects at work that are functional to accomplish work tasks, can induce motivation and productivity.^42, 43^ Therefore, exploring the relation between residents’ HVCCC attitudes and job demands and resources might help identifying which job characteristics are important to consider when promoting HVCCC behavior in the workplace.

Previous studies exploring the attitudes of physicians, administrators, patients, and learners toward HVCCC have typically focused on a single stakeholder group.^39, 44–47^ In this study, we aimed to perform a more comprehensive examination of the residency learning environment. Consequently, we surveyed residents, faculty, administrators, and patients regarding their attitudes toward HVCCC. We also investigated residents’ estimates of the HVCCC attitudes of other stakeholder groups and tested how the estimates differ from the embraced attitudes of these stakeholder groups. Furthermore, we examined what factors related to with attitudinal differences within each stakeholder group, and which job demands and resources related to residents’ HVCCC attitudes.

## METHOD

### Participants and Procedure

We conducted a cross-sectional survey (distributed between June 2017 and December 2018) of residents, faculty, administrators, and patients across the Netherlands. The Ethical Review Board of the Netherlands Association for Medical Education approved this study (no. NERB814, amendment no. NERB817) before launch.

### Study Context: the Dutch Healthcare System Compared With the US Situation

The Netherlands and the USA are among the ten highest healthcare expenditure per capita countries in the world.^48^ In the Netherlands, 12.9% of the gross domestic product was spent on healthcare in 2018. In the USA, this percentage was 17.8. The Dutch healthcare system largely relies on public resources, whereas the American healthcare system depends mainly on the private sector.^49^ In the Netherlands, residents are trained in the workplace under supervision by faculty and have internships within several non(academic) hospitals, which is similar to residency education in the USA. Administrators in both contexts attend to the financial concerns of healthcare organizations. For the patient population in the Netherlands, there is a uniform approach where every patient has insurance and relatively low out-of-pocket costs. In the USA, nearly 1 in 12 persons are uninsured,^50^ health insurance is much more variable, and out-of-pocket costs can be substantial.^51^

### Survey Instrument

Survey items included the Dutch version of the Maastricht HVCCC Attitude Questionnaire (MHAQ), which measures key dimensions of HVCCC^4^ on three subscales: (1) *high-value care*, the degree to which the respondent thinks physicians should be responsible for the provision of high-value care; (2) *cost incorporation*, the degree to which the respondent thinks physicians should integrate costs in daily clinical practices; and (3) *perceived drawbacks*, which reflects the respondent’s beliefs about potential negative consequences of HVCCC (see supplemental Appendix A for a description and example item for each subscale^52^). For the first two subscales, a higher score reflects a more positive attitude; for the third subscale, a lower score reflects a more positive attitude. Respondents indicated their extent of agreement using a four-point Likert scale (1 = strongly disagree, 4 = strongly agree).

Demographic items for residents and faculty addressed age, gender, hospital name, training region, clinical experience (in years), training location, and specialty (surgical, non-surgical, and supportive, see Supplemental Appendix B). Items for administrators included age, gender, hospital name, training region, and type of administrator. Items for patients included age, gender, hospital name, medical specialty visited, number of inpatient admissions and/or outpatient visits (average per year), estimated number of physicians treating the patient, and self-perceived health (rated using a seven-point scale, from 1 = very bad to 7 = very good).

The resident version of the survey also included two items asking respondents to estimate the HVCCC attitudes of other stakeholders. Specifically, we asked residents to indicate the extent to which faculty, administrators, and patients would agree with a central item from subscale 2 and subscale 3 using the same four-point Likert scale. We decided not to ask an item from subscale 1 because general guidelines in medicine indicate physicians should provide high-value care to patients.^53^

Finally, residents answered the job demands-resources questionnaire,^54^ a 40-item instrument that measures perceptions of five job demands, including work pressure, cognitive demands, emotional demands, role conflict, and hassles, and five job resources, including autonomy, social support, feedback, opportunities for development, and coaching (see supplemental Appendix C for a description and example item for each subscale). Residents responded to these items using one of two five-point Likert scales (either 1 = never, 5 = very often, or 1 = strongly disagree, 5 = strongly agree).

### Data Collection

We approached hospital education committees from all academic training regions (*N* = 8), after which these committees recruited residents and faculty in several hospital meetings and formal educational events. We also recruited residents and faculty via a digital newsletter of the Dutch “Bewustzijnsproject,” a project advocating HVCCC in postgraduate education.^2^ Authors F.S. and L.S. recruited administrators from several hospitals. We approached patients via patient platforms (e.g., patient panel of a hospital), via a health insurance company in the Netherlands, and before and after consults in hospitals (after gaining approval from the hospital).

All residents, faculty, and administrators answered the questionnaire online; patients also had the option to answer on hardcopy. All respondents received an information letter and signed an informed consent.

### Data Analysis

Descriptive summary statistics were reported as means with standard deviations or frequencies with percentages, as appropriate. We performed multiple imputations to deal with missing values.^55^ As participants were nested in hospitals, we used multilevel analyses to answer the research questions. We used multilevel ANOVA’s to compare the means of different stakeholders’ attitude scores on the subscales of the MHAQ. We assessed residents’ estimations of other stakeholders’ HVCCC attitudes and compared these with the actual HVCCC attitudes of these stakeholders, using multilevel independent *t* tests for the aforementioned two items. We used multilevel regression analyses to examine the relations between HVCCC attitudes scores and the measured independent variables. We used a backward elimination procedure, as we had not set a sequence of adding variables a priori.^56^ For all analyses, we estimated the population mean (μ) and accompanying standard deviation (SD). We calculated the regression slope using β, determined statistical significance at *p* < 0.05, and used confidence intervals (CI) when appropriate. Additionally, we calculated the effect size Hedges’ *g* (*g*) and considered an effect size above 0.8 as large.^57^ We conducted all analyses using IBM SPSS Statistics 25.0 for Windows (Armonk, NY: IBM Corp.).

## RESULTS

A total of 312 residents, 305 faculty members, 53 administrators, and 1046 patients responded to the survey (Supplemental Appendix D lists several features of each group). In total, 3,348 (8%) of 42,825 values were missing. Two hundred ninety-nine residents (96%), 297 faculty members (97%), and 53 administrators (100%) filled out all items in the MHAQ. Patients collectively answered 88% (27,554/31,380) of all MHAQ items; the remaining 12% (3,826/31,380) were missing completely at random.

### HVCCC Attitudes of the Different Stakeholder Groups

**Figure 1:**
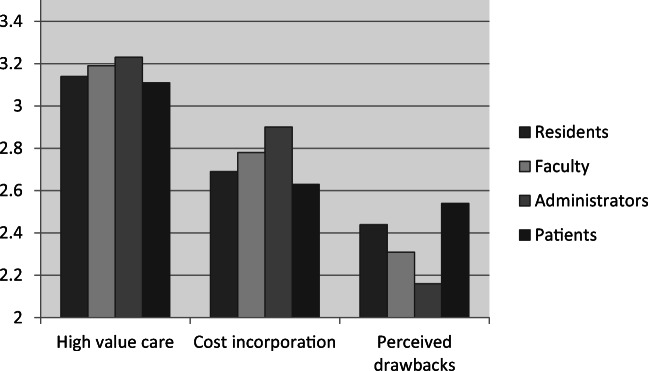
Overview of the subscale scores of the MHAQ per stakeholder group.

Figure 1 visualizes the mean scores per subscale and stakeholder group. Overall, faculty (abbreviated f) and administrators (abbreviated a) demonstrated more positive attitudes toward high-value care than patients (abbreviated p) (μ_f-p_ = 0.08, *p* = 0.002, *g* = 4.19; μ_f-p_ = 0.11, *p* = 0.03, *g* = 5.97) (Table 1). They were also more likely to agree physicians should integrate costs in daily clinical practices compared with residents (abbreviated r) (μ_f-r_ = 0.10, *p* = 0.005, *g* = 3.33; μ_a-r_ = 0.21, *p =* 0.001, *g* = 6.13) and patients (μ_f-p_ = 0.15, *p* = < 0.001, *g* = 6.54; μ_a-p_ = 0.27, *p* = < 0.001, *g* = 7.36). Residents endorsed more drawbacks to HVCCC than faculty (μ_r-f_ = 0.13, *p* = < 0.001, *g* = 4.91) and administrators (μ_r-a_ = 0.28, *p* = < 0.001, *g* = 8.20), but fewer drawbacks than patients (μ_r-p_ = − 0.10, *p* = 0.006, *g* = 5.33). Faculty, in turn, believed HVCCC had more potential drawbacks than administrators (μ_f-a_ = 0.15, *p* = 0.03, *g* = 4.29), while both faculty and administrators believed HVCCC has fewer drawbacks than patients (μ_f-p_ = − 0.23, *p* = < 0.001, *g* = 12.07; μ_a-p_ = − 0.38, *p* = < 0.001, *g* = 18.16).

**Table 1:** Overview of the estimated population means (μ), standard error of the mean (SEM) and estimated population mean differences (Δμ) between stakeholders.

**Estimated population mean differences**											
	μ	SEM	Compared to residents			Compared to staff physicians			Compared to administrators		
			Δμ	*p*	g	Δμ	*p*	g	Δμ	*p*	g
**High-value care** *Physicians should be responsible for the provision of high-value care*											
Residents	3.14	0.024	-	-	-	-	-	-	-	-	-
Staff physicians	3.19	0.025	0.049	0.084	2.04	-	-	-	-	-	-
Administrators	3.23	0.052	0.087	0.105	3.03	0.038	0.471	1.31	-	-	-
Patients	3.11	0.017	-0.027	0.272	1.59	-0.075	0.002†	4.19	-0.113	0.029^*^	5.97
**Cost Incorporation** *Physicians should incorporate costs in daily clinical practices*											
Residents	2.69	0.027	-	-	-	-	-	-	-	-	-
Staff physicians	2.78	0.027	0.097	0.005†	3.33	-	-	-	-	-	-
Administrators	2.90	0.062	0.210	0.001†	6.13	0.113	0.082	3.49	-	-	-
Patients	2.63	0.018	-0.055	0.053	2.94	-0.152	<0.001‡	6.54	-0.265	<0.001‡	7.36
**Perceived Drawbacks** *Believe that HVCCC has potential negative consequences*											
Residents	2.44	0.026	-	-	-	-	-	-	-	-	-
Staff physicians	2.31	0.027	-0.131	<0.001‡	4.91	-	-	-	-	-	-
Administrators	2.16	0.064	-0.279	<0.001‡	8.20	-0.148	0.033*	4.29	-	-	-
Patients	2.54	0.016	0.102	0.001†	5.33	0.233	<0.001‡	12.1	0.381	<0.001‡	18.2

*Note: g=Hedges’ g effect size*

* *p<0.05,* † *p<0.01,* ‡ *p<0.001*

### Residents’ Estimations of Other Stakeholders’ HVCCC Attitudes

Residents underestimated the attitudes of faculty regarding the physician’s duty to incorporate costs in daily practice (*p* = < 0.001, *g* = 8.03) and overestimated faculty’s attitude toward the drawbacks of HVCCC (*p* = < 0.001, *g* = 8.30) (Table 2). We found no significant differences between residents’ estimations on administrators’ attitudes and administrators’ embraced attitudes. Residents underestimated patients’ beliefs that HVCCC has drawbacks (*p* = < 0.001, *g* = 6.55); patients identified more drawbacks than residents were aware of.

**Table 2:** Model comparisons for each subscale, testing whether the addition of ‘stakeholder’ as a variable significantly improved the model.

Model	*N*_par_	-2 Log Likelihood	Comparison between model 1 and model 2			
			ΔX^2^	Δdf	F-ratio	*p*-value
High-value Care						
Model 1: Base model	3	1141.720				
Model 2: Base model + Stakeholder	6	1129.292	12.59	3	4.167	.007*
Cost Incorporation						
Model 1: Base model	3	1886.556				
Model 2: Base model + Stakeholder	6	1847.278	39.28	3	13.367	<0.001*
Perceived Drawbacks						
Model 1: Base model	3	2271.622				
Model 2: Base model + Stakeholder	6	2189.496	82.13	3	28.825	<0.001*

*Note: N*_*par*_ *= number of added degrees of freedom, each for every stakeholder. -2 Log Likelihood = statistical method used in multilevel analysis for estimating population parameters, displayed in smaller-is-better format*

*=p<0.05

### Factors Associated With Attitudinal Differences Within Each Stakeholder Group

Among *residents*, male gender and a non-surgical specialty were associated with more favorable attitudes toward high-value care (β = 0.09, 95% CI = 0.03, 0.16, *p =* 0.006; *p* = 0.006). However, male residents also endorsed more potential drawbacks (β = 0.13, 95% CI = 0.04, 0.21, *p* = 0.004), as did younger residents (β = 0.02, 95% CI = 0.03, 0.00, *p =* 0.01) and residents working in a surgical specialty compared with a supportive specialty (*p* = 0.03).

Among *faculty*, practicing in a supportive specialty was associated with more favorable attitudes toward high-value care compared with a surgical specialty (*p =* 0.002) and toward incorporation costs in daily practice compared with a surgical and non-surgical specialty (*p =* 0.02; *p =* 0.02). Older faculty members were more likely to believe physicians should incorporate costs (β = 0.01, 95% CI = 0.00, 0.008, *p =* 0.01) and less likely to endorse drawbacks of HVCCC (β = − 0.01, 95% CI = − 0.01, − 0.00, *p =* 0.001). Hospital region was significantly associated with faculty’s attitudes toward high-value care (*p =* 0.002) and cost incorporation (*p =* 0.004), but not with their beliefs about potential drawbacks. Among *administrators*, none of the variable measures predicted their attitudes toward HVCCC.

Among *patients*, more frequent hospital visits were associated with less favorable attitudes toward high-value care (β = − 0.00, 95% CI = − 0.01, − 0.00, *p* = 0.03) and cost incorporation (β = − 0.01, 95% CI = − 0.01, − 0.00, *p* = 0.04). A higher number of physicians treating the patients was associated with more favorable attitudes toward cost incorporation (β = − 0.03, 95% CI = − 0.05, − 0.00, *p* = 0.03). More positive perceptions regarding their own health condition were associated with more favorable attitudes toward high-value care (β = 0.03, 95% CI = 0.01, 0.06, *p* = < 0.001) and cost incorporation (β = 0.06, 95% CI = 0.03, 0.08, *p* = < 0.001), and less endorsement of drawbacks of HVCCC (β = − 0.07, 95% CI = − 0.10, − 0.05, *p* = < 0.001).

### Relation of Job Demands and Job Resources with Residents’ HVCCC Attitude

The more autonomy residents reported, the more positive their attitudes toward high-value care (β = 0.05, 95% CI = 0.01, 0.10, *p* = 0.03) and cost incorporation (β = 0.07, 95% CI = 0.02, 0.11, *p* = 0.008), and the fewer drawbacks they perceived (β = − 0.11, 95% CI = − 0.16, − 0.05, *p =* < 0.001). Higher perceived work pressure also related to more positive attitudes toward high-value care (β = 0.05, 95% CI = 0.02, 0.09, *p* = 0.004) and cost incorporation (β = 0.09, 95% CI = 0.04, 0.15, *p =* < 0.001). Opportunities for development positively related to high-value care (β = 0.07, 95% CI = 0.02, 0.12, *p* = 0.01), while supervisory coaching negatively related to high-value care (β = − 0.06, 95% CI = − 0.12, − 0.01, *p* = 0.02). Additionally, greater cognitive demands related to more favorable attitudes toward cost incorporation (β = − 0.09, 95% CI = − 0.16, − 0.02, *p* = 0.01), and the more emotional demands residents perceived, the more drawbacks they perceived (β = 0.08, 95% CI = 0.02, 0.14, *p* = 0.01). See Supplemental Appendix E for a complete overview of measured independent variables for each stakeholder group.

## DISCUSSION

This multisite survey study illuminates the attitudes of residents, faculty, administrators, and patients toward HVCCC. These groups represent key stakeholders in clinical learning environments where residents are expected to learn how to provide HVCCC.

Comparison of stakeholder attitudes toward HVCCC revealed several noteworthy differences, with faculty and administrators having the most positive attitudes toward HVCCC, and patients having the most negative. Faculty and administrators are challenged to steward healthcare resources in light of limited resources,^58^ act in accordance with societal developments (such as the Choosing Wisely campaign^59^), and adhere to evidenced-based medicine.^60^ Patients tend to focus predominantly on restoring their health^61^ and may lack information on how HVCCC can advance their health goals. Residents have to deal with this variation in attitudes. Their HVCCC attitudes are less favorable than faculty and administrators but more favorable than patients, which may reflect a natural developmental progression as residents transition from members of the lay public to members of the medical profession.

Because attitudes are internally held phenomena, residents must infer the attitudes of other stakeholders based on observations of their behavior, reports of others within the workplace, or impressions from the broader culture. We found residents underestimated the positive attitudes of faculty toward HVCCC but overestimated the positive attitudes of patients. These findings build on prior studies^62^ showing faculty’s thoughts on HVCCC are not always evident to residents. Faculty may display wasteful role-modeling behaviors^39^ which contribute to a hidden curriculum that contradicts their positive attitudes toward HVCCC. Additionally, residents may be unaware of their patients’ concerns or assume patients are better informed about the benefits of HVCCC than they actually are. Without this awareness, residents may fail to adequately address patient concerns, potentially compounding patients’ skepticism toward HVCCC and excluding patients from active participation in healthcare decisions.^63^

Physicians working in supportive specialties have fewer patient-related tasks,^64^ which might explain why these physicians see fewer drawbacks of HVCCC. Ageing faculty and residents were more favorable toward cost incorporation, which may reflect accumulating clinical experience and increased confidence in decision-making.^65^ Faculty in different regions displayed different attitudes, confirming the importance of workplace culture and regional practice patterns.^11, 15, 44^ The same association was not present for residents, perhaps reflecting shorter exposure to these potential influences in the learning environment. For patients, lower rates of their own health made them less open to HVCCC. This is in keeping with prior studies showing patients are primarily concerned about their own health,^61^ viewing HVCCC as a construct that could detract from or harm their care.

Regarding job characteristics, autonomy positively related to residents’ HVCCC attitudes. When residents feel they can make their own reasoned decisions, they might feel more confident and view HVCCC as an attainable challenge. Work pressure related to more favorable attitudes toward high-value care and cost incorporation. While high job demands can inhibit extracurricular activity,^66^ frontrunners of HVCCC may be overloading their tasks and thus experiencing more work pressure. Our findings indicate a relationship and not a causality, so work pressure may be a consequence of being more involved in HVCCC.

While stakeholders in the USA or other contexts may differ in their attitudes toward HVCCC, a similar pattern between stakeholders can be expected. Residents and faculty are similarly involved in patient care, administrators are responsible for managing finances, and patients are primarily focused on their health. In the USA, patients are generally receptive to discussing out-of-pocket costs,^67, 68^ but remain very wary of incorporating societal costs into care decisions.^67, 69^ This distinction between out-of-pocket and societal costs^70^ will most likely matter more to patients in the USA, where average out-of-pocket costs are 1.97 times higher than in the Netherlands.^71^ Replication of this study in other contexts can help clarify how stakeholder attitudes toward HVCCC may differ as a result.

### Implications

Our findings have several implications. First, it may be beneficial to forthrightly address the variation of HVCCC attitudes in the learning environment to help residents navigate the varying interests and perspectives they encounter. Senior faculty and specialty-specific teaching^72^ could play a role in this process. Second, the discrepancies between faculty’s attitudes and residents’ estimation of their attitudes suggest a need for faculty to more explicitly share their attitudes toward HVCCC and demonstrate these through their behavior, thereby setting good examples. Such role modeling is critical, as it represents a key mechanism by which social and occupational norms are transmitted.^18, 35, 37^ Patients also set norms for residents in postgraduate education,^73^ but are misjudged by residents. Training residents effective and empathic strategies for eliciting patients’ concerns and communicating the benefits of HVCCC may lead to more accurate perceptions and help boost patients’ confidence in such care. Finally, more autonomy at the workplace might contribute to more favorable HVCCC attitudes and behaviors, although further studies are needed to better understand the direction of associations with autonomy and work pressure, and explore potential trade-offs (e.g., related to patient safety or resident well-being).

### Strengths and Limitations

A strength of this study is that we surveyed multiple stakeholders from (non)academic hospitals geographically distributed over the Netherlands, supporting the study’s generalizability. We pursued several options recruiting respondents to reduce selection bias, and the large number of respondents per stakeholder group supports the representativeness of each sample. However, our study also has limitations. Despite the large sample, we cannot assure that participants represent the entire population, because of bias in who decided to fill out the questionnaire. This bias is hard to prevent, but we hope our large sample warrants good quality of data. We saw an acceptable but considerable rate of missing responses with patients, due to patients indicating not knowing the answer. As missing values were at random across all items, it was unlikely that this affected our results. Additionally, we are unable to report a response rate given the nature of our sampling strategy. We also did not include all potential stakeholders in the clinical learning environment, e.g., nurses might also affect residents’ thinking about HVCCC and could be involved in future research.

## CONCLUSION

Residents, faculty, administrators, and patients exhibit different HVCCC attitudes in the clinical learning environment, and residents misjudge the attitudes of faculty and patients. Residents may benefit from educators forthrightly addressing this variation, encouraging faculty and administrators to explicitly share and model their positive views, and providing empathic patient-centered strategies for communicating benefits of HVCCC.

## Electronic supplementary material

ESM 1 (DOCX 33 kb)

## Data Availability

The Dutch dataset collected during the current study is available from the corresponding author on reasonable request.
