# Supplementary material for: High-Value, Cost-Conscious Care Attitudes in the Graduate Medical Education Learning Environment: Various Stakeholder Attitudes That Residents Misjudge
Source: J Gen Intern Med. 2020 Nov 2;36(3):691–8. doi: 10.1007/s11606-020-06261-8 (PMC7947056; doi:10.1007/s11606-020-06261-8)
Supplement: Supplementary file 1 — (DOCX 33 kb) [file 11606_2020_6261_MOESM1_ESM.docx]

**Supplemental Appendix A**

Overview of the MHAQ, derived from Mordang et al. (53).

| **Construct** | **Number of items** | **Example item** |
| --- | --- | --- |
| High value care | 8 | Physicians should take a more prominent role in limiting use of unnecessary tests. |
| Cost Incorporation | 10 | Physicians should talk to patients about the costs of care when discussing treatment options. |
| Perceived Drawbacks | 7 | Practicing cost-conscious care will undermine patients’ trust in physicians. |

**Supplemental Appendix B**

Overview of the division of medical specialties in three categories.

Surgical: Cardiothoracic surgery, Gynecology and obstetrics, Surgery, Neurosurgery, Ophthalmology, Orthopedics, Otorhinolaryngology, Plastic surgery, Urology.

Non-surgical: Cardiology, Dermatology and venereology, Gastroenterology, Family medicine, Internal medicine, Pediatrics, Clinical geriatrics, Neurology, Geriatric medicine, Pulmonology, Psychiatry, Rheumatology, Rehabilitation medicine, Emergency medicine, Hospital medicine.

Supportive: Anesthesiology, Clinical biology, Clinical chemistry, Clinical genetics, Medical microbiology, Nuclear healthcare, Pathology, Radiology, Radiotherapy, Hospital pharmacy.

**Supplemental Appendix C**

Overview of Job demands and –resources measured in this study.

| **Construct** | **Description** | **Number of items** | **Example item** |
| --- | --- | --- | --- |
| *Job demands* |  |  |  |
| Work pressure | Level of pressure experienced at work. | 4 | Do you have too much work to do? |
| Cognitive demands | Level of memory and attentional resources required to be processed at work. | 4 | Do you regard your work as mentally very straining? |
| Emotional demands | Level of ‘strong’ feelings, such as grief, anxiety, anger and frustration at work. | 6 | Is your work emotionally demanding? |
| Role conflict | Level of incompatible demands that are put upon a person relating to their work. | 4 | I receive conflicting requests from two or more people. |
| Hassles | Level of irritating inconveniences. | 5 | I have to go through a lot of red tape to get my job done. |
| *Job resources* |  |  |  |
| Autonomy | Level of self-government at work. | 3 | Do you have control over how your work is carried out? |
| Social support | Level of perception and actuality that one is cared for at work. | 3 | Can you count on your colleagues to support you, if difficulties arise in your work? |
| Feedback | Level of information send to the individual about prior behavior at work. | 3 | I receive sufficient information about my work objectives. |
| Opportunities for  development | Level of (personal) development possibilities at work. | 3 | In my work, I can develop myself sufficiently. |
| Coaching | Level of supervisory support at work. | 5 | I feel valued by my supervisor. |

**Supplemental Appendix D**

| Characteristics | Residents | Faculty | Administrators | Patients |
| --- | --- | --- | --- | --- |
| N respondents | 312 | 305 | 53 | 1046 |
| N female respondents (%) | 200 (64.5) | 155 (50.8) | 26 (50) | 390 (37.3) |
| Age in years, M (SD) | 30.6 (2.8) | 45.9 (10.2) | 51.7 (8.8) | 61.8 (13.2) |
| Clinical experience in years, M (SD) | 4.1 (2.7) | 19.3 (9.4) | - | - |
| N of different hospitals | 30 | 26 | 6 | 59 |
| Medical specialty (%)*Surgical* *Non-Surgical*  *Supportive* | 308 (99.4)79 (25.5) 190 (61.3)  39 (12.6) | 299 (98.1)71 (23.3) 168 (55.1)  60 (19.7) | -- -  - | -- -  - |
| Type of administrator (%)*Department administrator* *Division administrator*  *Hospital administrator - Board level*  *Other Administrator* | - -  -  - | - -  - - | 17 (32.7) 13 (25)  7 (13.5)  15 (28.8) | - -  - - |
| Hospital visits per year, M (SD) | - | - | - | 4.5 (8.9) |
| Number of treating physicians, M (SD) | - | - | - | 1.6 (1.5) |
| Own perceived health quality**,** M (SD) | - | - | - | 5.1 (1.1) |

*Note: Abbreviations, M=mean, SD=standard deviation.*

**Supplemental Appendix E**

Overview of the adjusted regression model per stakeholder group.

|  | **Residents** |  | **Faculty** |  | **Administrators** |  | **Patients** |  |
| --- | --- | --- | --- | --- | --- | --- | --- | --- |
| Characteristic (DF) | Adjusted for covariates β (95% CI) | *F-*ratio | Adjusted for covariates β (95% CI) | *F-*ratio | Adjusted for covariates β (95% CI) | *F-*ratio | Adjusted for covariates β (95% CI) | *F-*ratio |
| **High-value Care** |  |  |  |  |  |  |  |  |
| Male Gender (1) | 0.09 (0.03, 0.16) | 7.681† | -0.00 (-0.07, 0.07) | 0.002 | -0.02 (-0.19, 0.15) | 0.045 | 0.04 (-0.01, 0.09) | 2.942 |
| Age (1) | 0.00 (-0.01, 0.01) | 0.084 | 0.00 (-0.00, 0.01) | 2.803 | 0.00 (-0.01, 0.01) | 0.001 | 0.03 (-0.02, 0.08) | 0.802 |
| Clinical experience (1) | -0.00 (-0.02, 0.01) | 0.129 | -0.00 (-0.01, 0.01) | 0.241 |  |  |  |  |
| Medical Specialty (3) |  | 3.899*^*^* |  | 4.773† |  |  |  |  |
| Training Site (7) |  | 1.506 |  | 0.863 |  |  |  |  |
| Region (7) |  | 0.158 |  | 3.275† |  | 1.834 |  | 1.008 |
| Type of Administrator (3) |  |  |  |  |  | 1.244 |  |  |
| Hospital Visits (1) |  |  |  |  |  |  | -0.00 (-0.01, 0.00) | 4.485*^*^* |
| Treating Physicians (1) |  |  |  |  |  |  | -0.01 (-0.03, 0.01) | 1.934 |
| Own Perceived Health (6) |  |  |  |  |  |  | 0.03 (0.01, 0.06) | 14.813‡ |
| *Job demands* |  |  |  |  |  |  |  |  |
| Work pressure (1) | 0.05 (0.02, 0.09) | 8.626† |  |  |  |  |  |  |
| Cognitive demands (1) | 0.00 (-0.06, 0.06) | 0.001 |  |  |  |  |  |  |
| Emotional demands (1) | -0.03 (-0.08, 0.03) | 1.024 |  |  |  |  |  |  |
| Role conflict (1) | -0.01 (-0.08, 0.05) | 0.186 |  |  |  |  |  |  |
| Hassles (1) | 0.02 (-0.04, 0.07) | 0.301 |  |  |  |  |  |  |
| *Job resources* |  |  |  |  |  |  |  |  |
| Autonomy (1) | 0.05 (0.01, 0.10) | 4.937*^*^* |  |  |  |  |  |  |
| Social support (1) | 0.02 (-0.03, 0.07) | 0.633 |  |  |  |  |  |  |
| Feedback (1) | -0.04 (-0.09, 0.02) | 1.978 |  |  |  |  |  |  |
| Opportunities for  development (1) | 0.07 (0.02, 0.13) | 6.791*^*^* |  |  |  |  |  |  |
| Coaching (1) | -0.06 (-0.12, -0.01) | 5.207*^*^* |  |  |  |  |  |  |
| **Cost Incorporation** |  |  |  |  |  |  |  |  |
| Male Gender (1) | 0.02 (-0.06, 0.10) | 0.209 | 0.04 (-0.03, 0.12) | 1.309 | 0.04 (-0.17, 0.25) | 0.157 | 0.04 (-0.03, 0.10) | 1.144 |
| Age (1) | 0.01 (-0.01, 0.02) | 0.680 | 0.00 (0.00, 0.01) | 6.301*^*^* | -0.00 (-0.01, 0.01) | 0.234 | 0.00 (-0.00, 0.00) | 2.929 |
| Clinical experience (1) | -0.00 (-0.02, 0.01) | 0.336 | -0.00 (-0.02, 0.01) | 0.443 |  |  |  |  |
| Medical Specialty (3) |  | 0.893 |  | 3.290*^*^* |  |  |  |  |
| Training Site (7) |  | 1.661 |  | 0.996 |  |  |  |  |
| Region (7) |  | 0.749 |  | 3.106† |  | 0.668 |  | 1.933 |
| Type of Administrator (3) |  |  |  |  |  | 0.871 |  |  |
| Hospital Visits (1) |  |  |  |  |  |  | -0.00 (-0.01, -0.00) | 4.287*^*^* |
| Treating Physicians (1) |  |  |  |  |  |  | -0.03 (-0.05, -0.00) | 4.804*^*^* |
| Own Perceived Health (6) |  |  |  |  |  |  | 0.06 (0.03, 0.08) | 14.181‡ |
| *Job demands* |  |  |  |  |  |  |  |  |
| Work pressure (1) | 0.09 (0.04, 0.14) | 12.758‡ |  |  |  |  |  |  |
| Cognitive demands (1) | -0.09 (-0.16, -0.02) | 6.596*^*^* |  |  |  |  |  |  |
| Emotional demands (1) | -0.01 (-0.08, 0.07) | 0.033 |  |  |  |  |  |  |
| Role conflict (1) | 0.04 (-0.03, 0.11) | 1.325 |  |  |  |  |  |  |
| Hassles (1) | -0.04 (-0.11, 0.02) | 2.015 |  |  |  |  |  |  |
| *Job resources* |  |  |  |  |  |  |  |  |
| Autonomy (1) | 0.06 (0.02, 0.11) | 7.164† |  |  |  |  |  |  |
| Social support (1) | 0.04 (-0.02, 0.09) | 1.900 |  |  |  |  |  |  |
| Feedback (1) | -0.01 (-0.07, 0.06) | 0.026 |  |  |  |  |  |  |
| Opportunities for  development (1) | 0.00 (-0.07, 0.07) | 0.001 |  |  |  |  |  |  |
| Coaching (1) | -0.04 (-0.10, 0.02) | 1.667 |  |  |  |  |  |  |
| **Perceived Drawbacks of HVCCC** | |  |  |  |  |  |  |  |
| Male Gender (1) | 0.13 (0.04, 0.21) | 8.616† | 0.04 (-0.05, 0.13) | 0.601 | -0.06 (-0.26, 0.14) | 0.376 | 0.02 (-0.04, 0.09) | 0.576 |
| Age (1) | -0.02 (-0.03, -0.00) | 6.642*^*^* | -0.01 (-0.01, -0.00) | 10.664† | 0.01 (-0.01, 0.02) | 0.867 | 0.00 (0.00, 0.01) | 5.923*^*^* |
| Clinical experience (1) | 0.01 (-0.01, 0.03) | 1.143 | 0.00 (-0.01, 0.02) | 0.243 |  |  |  |  |
| Medical Specialty (3) |  | 3.053*^*^* |  | 1.803 |  |  |  |  |
| Training Site (7) |  | 1.389 |  | 1.486 |  |  |  |  |
| Region (7) |  | 1.383 |  | 1.610 |  | 1.051 |  | 0.286 |
| Type of Administrator (3) |  |  |  |  |  | 0.924 |  |  |
| Hospital Visits (1) |  |  |  |  |  |  | -0.00 (-0.00, 0.00) | 0.044 |
| Treating Physicians (1) |  |  |  |  |  |  | 0.02 (-0.01, 0.04) | 2.136 |
| Own Perceived Health (6) |  |  |  |  |  |  | -0.07 (-0.10, -0.05) | 26.599‡ |
| *Job demands* |  |  |  |  |  |  |  |  |
| Work pressure (1) | -0.02 (-0.07, 0.03) | 0.473 |  |  |  |  |  |  |
| Cognitive demands (1) | 0.04 (-0.04, 0.12) | 0.953 |  |  |  |  |  |  |
| Emotional demands (1) | 0.08 (0.02, 0.14) | 6.340*^*^* |  |  |  |  |  |  |
| Role conflict (1) | -0.04 (-0.12, 0.05) | 0.746 |  |  |  |  |  |  |
| Hassles (1) | 0.04 (-0.03, 0.11) | 1.261 |  |  |  |  |  |  |
| *Job resources* |  |  |  |  |  |  |  |  |
| Autonomy (1) | -0.11 (-0.16, -0.05) | 15.380‡ |  |  |  |  |  |  |
| Social support (1) | -0.03 (-0.09, 0.04) | 0.696 |  |  |  |  |  |  |
| Feedback (1) | -0.00 (-0.07, 0.07) | 0.007 |  |  |  |  |  |  |
| Opportunities for  development (1) | 0.01 (-0.06, 0.08) | 0.111 |  |  |  |  |  |  |
| Coaching (1) | -0.03 (-0.10, 0.03) | 0.922 |  |  |  |  |  |  |

*Note: ^*^=Significant at p=.05,* †*=significant at p=.01,* ‡*=significant at p<.001. DF=degrees of freedom.*
